# Omics for the Improvement of Abiotic, Biotic, and Agronomic Traits in Major Cereal Crops: Applications, Challenges, and Prospects

**DOI:** 10.3390/plants10101989

**Published:** 2021-09-23

**Authors:** Balwinder Kaur, Karansher S. Sandhu, Roop Kamal, Kawalpreet Kaur, Jagmohan Singh, Marion S. Röder, Quddoos H. Muqaddasi

**Affiliations:** 1 Everglades Research and Education Center, University of Florida, 3200 E. Palm Beach Rd., Belle Glade, FL 33430, USA; bkaur@ufl.edu; 2 Department of Crop and Soil Sciences, Washington State University, Pullman, WA 99163, USA; k.sandhu@wsu.edu; 3 Leibniz Institute of Plant Genetics and Crop Plant Research (IPK), Corrensstraße 3, 06466 Stadt Seeland, Germany; kamal@ipk-gatersleben.de (R.K.); or marionroder61@gmail.com (M.S.R.); 4 Department of Agricultural, Food and Nutritional Science, University of Alberta, Edmonton, AB T6G 2P5, Canada; kawalpre@ualberta.ca; 5 Division of Plant Pathology, ICAR-Indian Agricultural Research Institute, New Delhi 110012, India; dhillonjagmohansingh@gmail.com

**Keywords:** cereals, omics, genomics, transcriptomics, proteomics, metabolomics, phenomics

## Abstract

Omics technologies, namely genomics, transcriptomics, proteomics, metabolomics, and phenomics, are becoming an integral part of virtually every commercial cereal crop breeding program, as they provide substantial dividends per unit time in both pre-breeding and breeding phases. Continuous advances in omics assure time efficiency and cost benefits to improve cereal crops. This review provides a comprehensive overview of the established omics methods in five major cereals, namely rice, sorghum, maize, barley, and bread wheat. We cover the evolution of technologies in each omics section independently and concentrate on their use to improve economically important agronomic as well as biotic and abiotic stress-related traits. Advancements in the (1) identification, mapping, and sequencing of molecular/structural variants; (2) high-density transcriptomics data to study gene expression patterns; (3) global and targeted proteome profiling to study protein structure and interaction; (4) metabolomic profiling to quantify organ-level, small-density metabolites, and their composition; and (5) high-resolution, high-throughput, image-based phenomics approaches are surveyed in this review.

## 1. Introduction

**Figure 1:**
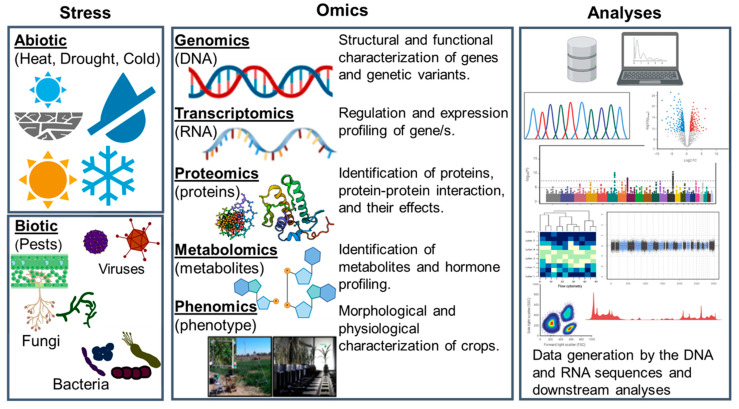
An overview of the use of omics in cereal crop improvement. Abiotic and biotic stresses, for example, can be countered by the application of a set of omics technologies that result in a large-scale data production, which later on needs to be analyzed via different computational and analyses pipelines. Images in the figure are created with BioRender.com.

Our better understanding of the genetic and molecular principles in major cereal crops, including rice (*Oryza sativa* L.), sorghum (*Sorghum bicolor* L.), maize (*Zea mays* L.), barley (*Hordeum vulgare* L.), and bread wheat (*Triticum aestivum* L.), have resulted in a continuous growth in their production (https://www.fao.org; accessed 13 August 2021). The genetic gain in cereal crops can be ascribed to their superior breeding as well as agronomic and crop-protection management practices. Omics as a set of technologies that encompass genomics, transcriptomics, proteomics, metabolomics, and, most recently, phenomics has contributed fundamentally to the breeding part. As summarized in Figure 1, the use of omics has led to extensive data production and, consequently, enhanced our understanding of the (1) morphology and growth patterns, (2) genetical underpinnings of various qualitative and quantitative traits, (3) the expression of different genes, and (4) mechanisms as to how the complex interactions among genes, proteins, and metabolites contribute to the resulting phenotype.

Omics applications—because they often result in the production of massive data points—have also resulted in advancement of analytical tools, large-scale computational facilities, and high-throughput data analyses pipelines [1]. From a purely applied breeding standpoint, the use of omics has and continues to contribute to (1) increased genetic gain and (2) shortened life of a breeding cycle. For example, the use of high-throughput phenotyping and genotyping platforms contribute not only to higher accuracy of the genetic mapping of valuable traits but also predicted genetic/breeding values of the cereal crops. The use of marker-assisted or genome-wide selection thus results in improving the breeding pipelines. On the other hand, transcriptomic, proteomic, and metabolomic data inform us about the expression patterns and protein profiling of the identified loci as well the metabolic pathways of the genes.

Here, with the objective to provide the reader an overview of the omics technologies and their uses, we summarize five different omics technologies. In every omics section, we provide background as well as summarize the cutting-edge techniques/platforms. We concentrate on five leading above-mentioned cereal crops and give a general overview as to how these technologies have helped in addressing some abiotic and biotic stresses.

## 2. Cereal Genomics: Evolution from Sparse Genetic Markers to Whole-Genome Sequencing

Identification of molecular markers—the observable polymorphisms within a given DNA sequence among the individuals of a population—laid the foundations of modern genomics. In the 1980s, the detection of restriction fragment-length polymorphisms (RFLPs) and their subsequent association with several agronomically important traits forecasted the promises of genomics to improve the genetic gain per unit time. Later, many other marker systems—most notably microsatellite or simple sequence repeats (SSRs)—were used to map the quantitative trait loci (QTL). Nevertheless, albeit an excellent use of SSRs in applied breeding, these systems were time and cost inefficient and low throughput. For example, the first SSR map of bread wheat harbored only 279 loci [2]. Most economically important traits, for example, grain yield, disease resistance, and grain protein content, are polygenic, i.e., they are controlled by the concerted action of several small- to medium-effect genetic loci [3]. Therefore, sparse genetic-linkage maps harboring a limited number of genetic loci become inefficient for improving highly complex or quantitative traits mainly because of the absence of trait-linked loci.

Detection of single nucleotide polymorphisms (SNPs)—the smallest unit of DNA polymorphism—provides an opportunity to survey virtually millions of sites within the DNA of a species. Thus, it has become a choice marker platform. High throughput, high efficiency, reproducibility, and low cost per data point of SNPs have enabled large-scale germplasm evaluations in many cereal crops and, consequently, have resulted in the almost complete replacement of RFLPs or SSRs [4]. Major methods for SNP detection in cereals include array-based genotyping and genotyping by sequencing. Several sequencing technologies are available for both forms of SNP detection [4]. Thus, high-density SNP genotyping is invaluable for identifying the genetic underpinning of economically relevant traits and laying the foundation of whole-genome sequencing (WGS).

**Figure 2:**
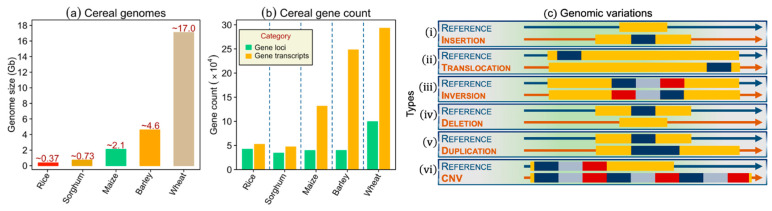
Cereal genome features. (**a**) Approximate genome size in Gb, (**b**) genetic loci and the transcripts harbored by top cereal crops, and (**c**) types of major genomic variants. The data for the subfigures (**a**,**b**) is mainly gathered from the “Phytozome” website (https://phytozome-next.jgi.doe.gov/ accessed 5 June 2021), and the references to each sequence are given in the main text.

While WGS for hundreds of animal species had already been reported, it is only recently that the genomes of major cereal crops were fully sequenced (available online at NCBI [5]). The technological innovations coupled with international collaborative consortia efforts have led to the construction of cereal genome assemblies that may be used for several applied genetic purposes, such as genome-wide scans for genes controlling important traits. However, sequencing the cereal genomes has been challenging mainly because of their large sizes and abundance of repetitive sequences [6]. Three of the major cereals, namely rice, sorghum, and maize, have been very well-sequenced using various technologies—the foremost reason being their diploid nature and small genomes. For example, primarily because of limited genome size and diploid nature, rice became a model choice for the WGS, and in 2002, working genome drafts of domesticated rice subspecies (ssp. *japonica* and *indica*) were published [7,8]. Following rice, seven years later, sorghum and maize genomes were published [9,10]. Barley and bread wheat—among the most important members of the Triticieae tribe—were difficult to sequence mainly because of their large genome sizes (Figure 2a,b). The first draft assembly of barley (cultivar Morex) was assembled based on genome-zipper, whole-genome shotgun contigs, and bacterial artificial chromosomes (BAC) clones [11]. Bread wheat genome assembly was complicated primarily because of its complex polyploid nature with many chromosomal duplications, rearrangement, and the presence of a high percentage of repetitive sequence (~80%) [12]. In 2014, the first wheat genome draft assembly based on chromosome-sorted whole-genome shotgun sequences was released by the IWGSC [12]. More recently, in 2018, IWGSC released a reference sequence of wheat cultivar Chinese spring (RefSeq v1.0). The RefSeq v1.0 resulted in 94% coverage of the wheat genome and contained 107,981 high-confidence gene models that could be used for constructing complex gene networks and pathways [13].

### 2.1. Genome Sequencing Technologies

Sequencing technologies have emerged to reveal the valuable information hidden in plant genomes. First-generation sequencing technologies, e.g., Sanger sequencing and Maxam–Gilbert chemical cleavage, marked the start of the genomic era. Sanger sequencing remains beneficial to date, especially where high-throughput sequencing is not required, such as verifying the plasmid construct or a PCR product. Nevertheless, the need for high-throughput information for the large plant genomes at lower cost triggered the development of the second-generation sequencing technologies that can be grouped into two categories, namely sequencing by hybridization and sequencing by synthesis. Examples of the second-generation sequencing technologies include 454 Pyrosequencing, Ion Torrent, and Illumina Tech. Both first- and second-generation sequencing technologies generate short-reads ranging from 50 to 1000 bp fragments, making them suitable for resequencing projects, SNP calling, and targeted sequencing of the short amplicons.

Short-read sequencing is, however, not suitable for large-scale projects: this necessitated third-generation platforms, such as large fragment single-molecule technologies. The third-generation sequencing platforms include PacBio (or single molecular real-time; SMRT) and Oxford Nanopore sequencing. In principle, both short- and long-read technologies can be used for genome assembly. However, the short-read technologies usually lead to incomplete assemblies (draft) and, hence, missed sequencing, loss of gene information, and reduction in the accuracy of the downstream analyses, such as detection of structural variations (SVs) [14]. Highly accurate long reads, on the other hand, generate overlapping sequences that (1) allow generation of complete genome assemblies, (2) span large structural variations, and (3) sequence extreme GC regions that otherwise cannot be sequenced with the short-read sequencing technologies. These next-generation sequencing (NGS) platforms aid in resequencing as well as *de novo* genome assembly that can be used to find genomic variants either by aligning the draft genome to a reference genome or assembling a new genome sequence from the overlapping reads.

Nonetheless, the long-read sequencing by both PacBio and Oxford Nanopore technologies produces reads that are still not sufficient to cover some large, repetitive, and complex genomic regions. To overcome the assembly problems, Hi-C sequencing and optical mapping can be used. Hi-C is a recent version of the chromosome conformation capture (3C) with NGS techniques. The 3C technology was adopted in plants in 2009. In Hi-C technique, the contact probability of the two loci that are closer in the 3D-nuclei space is higher compared to the loci that are far from each other. By using the Hi-C principle, physical mapping data was generated in wheat and barley that was utilized for genome assembly projects [15]. The methodology advancement led to chromosome conformation capture on chip (4C) and chromosome conformation capture carbon copy (5C) technologies. Tethered chromosome conformation capture (TCC), capture Hi-C, in situ Hi-C, and single-cell Hi-C are variants of the Hi-C technology developed to enhance the signal to noise ratio [15]. Even though the cost of Hi-C is low, the technology suffers from sequencing biases that make it error prone. Moreover, Hi-C can also lead to misassemblies, such as scaffold misplacement and false inversions.

In contrast to Hi-C, a light microscope-based technology is used in optical mapping to physically locate specific enzyme or sequence motif. These enzymes and motives are then used to produce DNA sequence fingerprints. The DNA molecule is fluorescently labelled by selected enzymes, and later on, the optical maps are produced using the images of the fluorescent signal patterns from the labelled enzymes. Generally, the optical map is larger than the reads produced by both short- and long-read sequencing techniques, and the average molecular length of an optical map is ~225 kb. Therefore, these optical maps span genomic regions that are challenging to resolve by other DNA sequencing technologies and are used for genome assembly improvement, to identify large SVs, and in haplotype phasing [16]. Most recently, optical mapping was used to refine the assembly of the wheat genome by producing RefSeq v2.1 [17].

### 2.2. Types of Genomic Variants: Applications in Genetics and Breeding

Every applied plant breeding program’s success hinges on artificial selection—a process that involves the selection, preservation, and propagation of plants possessing the most desirable characters from a diverse population. Artificial selection is determined by the considerable genotypic variation in a population that results in the high heritability of the traits under selection. Variation and, in this case, genotypic variation is a type of variation that is directly ascribed to the genetic differences among individuals of a given population. Therefore, genomics can help to improve crops because it assesses the genetic or genomic polymorphisms among individuals.

Differences in the plant species arise from SNPs to larger SVs within its genome. These SVs include insertions, deletions, copy number variations (CNVs), and many more. Some of these variants are described below as well as in Figure 2c.

#### 2.2.1. Single Nucleotide Polymorphisms

With high-throughput and cost-effective sequencing, it is relatively easier to discover millions of SNPs in a plant species. SNPs are frequently found in the genomes and primarily lay the foundations of the genetic diversity among individuals of a given population. Both coding and non-coding regions of the genome can harbor SNPs that can consequently alter the expression profile. Therefore, uncovering the functional SNPs in gene/s and finding their effects on the phenotype can help to understand the gene function and, subsequently, its product.

Rice—along with other cereals—presents one example to showcase SNPs’ abundant nature. Approximately 20 million SNPs were discovered by aligning the sequences of ~3000 rice genomes against the Nipponbare reference sequence [18]. SNPs’ use in cereal breeding programs is indisputable where high-density genotyping resulted in associations of SNPs with several traits of central agronomic value via linkage mapping and genome-wide association studies (GWAS; described later in this section). In sorghum, a large SNP database SorGDS is available that can be exploited for genetic studies [19]. Similarly, barley tool BRIDGE can be exploited for SNP discovery [20]. Recently, Sun et al. [21] presented a comparison of different wheat arrays for SNP discovery.

#### 2.2.2. Variants Apart from SNPs

While SNPs are an essential source for identifying and mapping traits of interests, studies show that “only” SNPs do not represent all the genomic variation that contributes to the resulting phenotype, and therefore, other variants, for example, SVs—that may be up to 1-kb long—play an essential role as well. Inversion, translocation, deletion, insertion, and CNVs all come under the umbrella of SVs. Maize is the first cereal in which hundreds of SVs were identified. However, later, this number was found to be underestimated, and efforts were initiated to discover more SVs among higher eukaryotes.

The studies of SVs were recently accelerated in the crop plants primarily due to the reference genome sequence generation. Based on the sequence similarity at the DNA breakpoints, SVs are formed mainly by two mechanisms: non-homologous end-joining (NHEJ) and non-allelic homologous recombination (NAHR) [22]. Apart from these mechanisms, transposons also generate SVs. In general, SVs can be detected mainly by three methods: (1) re-sequencing, (2) the de novo assembly, and (3) the pangenome assemblies. The resequencing approach mainly identifies CNVs and presence-absence variations (PAVs), whereas the de novo approach—along with CNVs and PAVs—also identifies inversions. Nevertheless, the resequencing approach remains the preferred approach to detect the SVs due to its low cost and lack of de novo assembly generation for each variety under investigation. The CNVs arise from the unbalanced DNA modifications that lead to the variable number of copies of a specific DNA sequence [23]. CNVs may vary from 1- kb to several Mbs. Studies show that, along with SNPs and InDels, CNVs are key contributors to intra-species genetic variation. The PAVs can be considered as the extreme form of the CNVs. In PAV, a genomic sequence is present in one individual and absent from the other. In the past few years, SVs affect several traits in different cereals. For instance, 17.1-kb tandem duplication of *GL7* locus in rice leads to an increase in the grain size [24], CNVs of *Vrn-A1* and *Ppd-B1* affect the flowering time in wheat [25], a 7-bp deletion on *HvGA20ox2* gene reduces the plant height and delay flowering time in barley [26], and a complex tandem repeat array inserted upstream of the *mlo-11* locus confers resistance to powdery mildew in barley [27].

### 2.3. Genetic Mapping

Several statistical methods can be employed to link the polymorphism to the traits under investigation—most common of which are regression analyses. In cereal crops, polymorphisms or variations among individuals can be (1) artificially generated via crossing different parents and (2) surveyed in a natural population consisting of a set of elite lines, or gene bank accessions, etc. In the following, we provide the most common methods to link the genetic polymorphisms to the traits under investigation.

#### 2.3.1. Genome-Wide Linkage Mapping

Genome-wide linkage mapping (GWLM) refers to mapping the QTL in mostly artificially created segregating populations. Many traits of economic importance, such as grain yield, stress tolerance, and disease resistance, are of quantitative nature [3]. Therefore, segregating populations harboring virtually hundreds of individuals are required to dissect the genetic nature of a quantitative trait. Different types of segregating populations, such as F_2_ population, recombinant inbred lines (RILs), doubled haploid (DH) population, heterogeneous inbred family (HIF), near-isogenic lines (NILs), advanced intercross recombinant inbred lines (AI-RIL), backcross inbred lines, and multiparent advanced generation intercross (MAGIC), are developed based mainly on the available resources and research objectives. These segregating populations are mainly based on crosses between contrasting parents, resulting in a limited genetic diversity. GWLM is the most commonly used method to detect genes underlying essential traits. Nevertheless, resources and time to develop these mapping populations coupled with a narrow genetic base plus low allelic richness and mapping resolution are some drawbacks of GWLM.

#### 2.3.2. Genome-Wide Association Studies

Genome-wide association studies (GWAS) take advantage of the long history of recombination events in the diverse natural population to dissect the genetic nature of a trait. The use of natural population overcomes the constraints of the GWLM as it increases the mapping resolution and reduces the research time [28]. GWAS was initially used to study the complex traits in humans, and then, it was adopted for animals and some model organisms. In the last decades, with the improvements in genotyping techniques, decreased cost of sequencing, and robust statistical methods, researchers have adopted the GWAS for dissecting the genetic architecture of complex traits in plants. GWAS identifies marker-trait associations (MTA) that can be attributed to the strength of linkage disequilibrium (LD) between polymorphic markers across a set of diverse germplasm. In a nutshell, GWAS analysis is performed to evaluate each genotyped marker’s association with a trait of interest that has been scored across a diverse natural population. GWAS analysis can be used to study both qualitative and quantitative traits. Several aspects must be considered for starting the research, such as selection of genotyping platform, sample or population size and structure, statistical analyses, and correction for multiple testing (e.g., Bonferroni correction, false discovery rate, etc.). Although not an exhaustive list, Table 1 enlists the use of GWLM, GWAS, or both for some agronomically important traits for cereal crop improvement in the last few years.

### 2.4. The Study of Species-Level Variations via Pangenomes

The pangenome aims to discover genic PAVs within a species [54]. A pangenome contains a core genome, i.e., genomic sequences present in all the individuals of a species and a variable genome, i.e., genomic sequences present in some individuals. The first step to establish a pangenome in any crop species is selecting a diverse set of genotypes, including domesticated and wild progenitors, for sequence assembly. It is also wise to choose genotypes of high breeding or genetic value to increase the pangenome’s importance for future breeding programs. Genotypes belonging to secondary and tertiary gene pools of a particular species are added to form a genus-level pangenome. The reference-quality genomes are then generated for the small set of accessions and aligned to a reference genome to detect the SVs. The k-mers present in the SVs are extracted and determined in the form of short-read data from a diversity panel to genotype the underlying SVs, and the matrices of the k-mers count are used as biallelic markers for the GWLM or GWAS [55]. Pangenome has already been established in various cereal crops, such as rice [56], wheat [57], and barley [54]. For example, a pangenome of 20 assemblies was constructed in barley, single-copy k-mers from the structural variants in these 20 assemblies were detected, and a k-mer abundance matrix was used to perform the GWAS for lemma adherence [54].

### 2.5. Challenges and Prospects in Crop Genomics

In the past, WGS efforts were hindered mainly by the (1) extensive and repetitive genome sequences of the cereals and (2) the absence of current technologies and algorithms that are robust and exact in generating and assembling the large and correct sequences. Therefore, this has perhaps been the most crucial reason why considerable international consortia efforts were required. Although large-scale genome sequence production and assembling are currently costly, with continuous innovation in technologies, future large-scale, reference-quality genome assemblies will be easier mainly because of the small cost-outcome differential. It can be safely speculated that the construction of genome assemblies will continue to the point where the difference between whole-genome genotyping and WGS will be negligible [6]. With the improvements in sequencing and computing facilities, the production per unit of input will be improved, which will be beneficial for cereal geneticists and breeders. As described elsewhere, robust QTL mapping and gene cloning hinge on dense genetic/physical maps’ availability. Advances in genomics will help in fast and accurate mapping of the traits. Additionally, with the availability of the dense marker information, the methods of prediction of genotypic or breeding value will become more efficient to improve the genetic gains per unit time and cost.

## 3. Cereal Transcriptomics

Genomics provide details about the genetic content and existing variation of an organism. However, genomics does not inform about the portion of the expressed genome and level to which a gene is expressed. It is important to note that only 1–2% of the genome of an organism is expressed that encodes for functional or regulatory proteins. The extensive study of this expressed genome is provided by transcriptomics that measures the expression of genes in an organism in different conditions, tissues (spatial transcriptome) and time points (temporal transcriptome).

### 3.1. Transcriptomics Techniques

The first attempt to study RNA transcripts was made in the 1970s, when mRNA libraries of silk moths were converted to cDNA using reverse transcriptase [58]. Later, in the 1980s, Sanger sequencing was used to sequence the RNA transcripts, called Expressed Sequence Tags (ESTs) [59]. EST was used as a technique to determine the gene content of an organism. Later, RNA transcript quantification was also performed using various techniques, such as northern blotting and qRT-PCR [60]. However, these techniques do not cover the entire transcriptome but only a small part of it. In 1995, the first method developed and used for transcriptomics was sequencing-based and wascalled Serial Analysis of Gene Expression (SAGE) [61].

SAGE methodology involves preparing a short sequence tag (10–14 bp) from each transcript’s unique position which can be used to identify the transcript. Sequence tags are linked together to form long serial molecules: these molecules are then cloned and sequenced. To check the expression of a specific gene, a total number of tags are counted. Quantification of a particular tag provides the expression level of the corresponding gene. SAGE can also help to identify new genes expressing in a tissue or under specific conditions [61].

Later, well-defined techniques for example microarrays, massively parallel signature sequencing (MPSS), and RNA-seq that provide high-throughput transcriptomics data came into existence. Microarray quantifies a set of the RNA transcripts by their hybridization to complementary probes fixed on a platform. It was used to assay thousands of genes with a low cost per unit gene. Advancements in designing arrays and fluorescence detection systems have boosted the sensitivity and accuracy of this technique. A microarray consists of several probes on a solid platform, i.e., a glass or a silicon chip. The fluorescent-labeled transcripts then hybridize on these chips to complement the probes. The amount or intensity of fluorescence at each probe quantifies the respective transcript [62]. Microarrays are broadly of two categories: low-density spotted array and high-density probe array. Low-density spotted arrays use large probes and various fluorophores for test and control, whereas high-density probe arrays have higher resolution and use a single fluorophore for the test [63]. Initially, Affymetrix (Santa Clara, CA, USA) Gene chip array developed a high-density array, and later, Nimble Gen developed a more advanced high-density array by mask-less photochemistry. Other commercially available microarray platforms are Agilent, Exiqon, and Miltenyi, etc. Even though this technique is efficient in revealing the transcripts in an organism, it requires prior knowledge of ESTs and an organism’s genome assembly so that probes could be designed to generate the chip.

MPSS is a sequencing-based approach used to analyze gene expression by quantifying mRNA transcripts present in the samples. MPSS uses a 17–20 bp signature sequence adjacent to the 3′-end of mRNA to identify mRNA. Each signature sequence is first cloned onto microbeads. This technique ensures that only one type of DNA sequence is on a microbead. The microbeads are arrayed in a flow cell for sequencing and quantification. Each signature sequence (MPSS tag) in a MPSS dataset is analyzed, compared with all other signatures, and all identical signatures are counted. The expression level of any single gene is calculated by dividing the total number of signatures for that gene present in the samples with all signature sequences identified.

**Figure 3:**
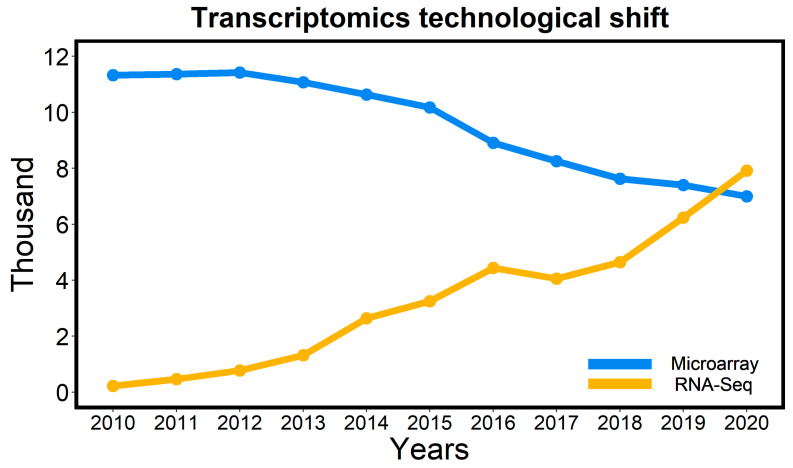
The number of publications of “Microarray” and “RNA-Seq” for the last ten years depicts the use and technological shift in transcriptomics. (Source: PubMed).

RNA-seq is defined as sequencing the mRNA transcripts of an organism by using NGS platforms. High-throughput sequencing platforms have highly reduced the cost of sequencing and increase the level of accuracy. New sequencing platforms, such as Roche 454, Illumina, SOLiD, Pac Bio, and Nanopore (compared in Table 2), have aided the RNA-seq technique to provide extensive genome coverage [64,65]. RNA-seq provides a tremendous amount of information about the genes present and activation of these genes at a particular time point under specific conditions. In recent years, the availability of NGS technologies has boosted RNA-seq over microarray technique, illustrated by the number of publications in the last ten years (Figure 3).

Only the mRNA transcripts are sorted out from different kinds of RNAs for RNA-seq. The mRNAs with poly-A tail are separated out from the whole RNA by poly-A tail-specific probes. Small RNAs are removed based on their size by using gel electrophoresis. The mRNAs are fragmented as per the read-length limit of the sequencing technology through hydrolysis or sonication. The selected mRNA is used to synthesize cDNA, which could be amplified if the amount is not sufficient and finally used as reads for sequencing through NGS platforms [66]. Presently, NGS techniques, such as PacBio and Oxford Nanopore, directly sequences RNA without conversion into cDNA. It is better than previous sequencing techniques, as it detects the modified bases, which were otherwise masked during cDNA synthesis, and prevents the biases introduced during the cDNA-amplification step. The number of reads and amount of coverage of the genome determines the sensitivity and accuracy of RNA-seq. The Encyclopedia of DNA Elements (ENCODE) recommends 70× coverage for standard RNA-seq and even 500× coverage for rare transcripts [67].

NanoString is another newly developed hybridization-based method that uses two probes for a target transcript, one capture probe (biotin labeled), and another reporter probe (fluorescent barcode-labeled). The capture probe locks the transcript to a solid surface by biotin-avidin binding, whereas fluorescent barcode-labeled reporter probe hybridizes with specific mRNA transcript. The NanoString nCounter analysis system is used to quantify the immobilized mRNA transcripts by their specific barcode. The NanoString has its high utility in targeted transcriptomic studies. The edge of NanoString over NGS based tools is that it does not require library preparation, enzymes, and processing.

### 3.2. Transcriptomics to Study Abiotic Stress Tolerance in Plants

With an increase in the whole-genome transcriptomic studies in plants, the genes related to stress response, downstream signaling, and synthesis of stress response molecules are undermined [68]. A plethora of information on transcriptomics of cereals crops, such as rice, sorghum, maize, barley, and wheat, are available. This information has provided insight into the coordination of different biological processes in various plant tissues under stress conditions [69]. The study of drought stress during the flowering or fruiting stage of the plant gives information about the reproductive system’s interaction, hormone signaling, and metabolic pathways. Table 3 highlights the use of microarray and RNA-seq techniques in different crops to identify differentially regulated genes during various abiotic stress conditions.

The comparative transcriptome analysis between drought-tolerant and susceptible cultivars indicates candidate genes and the mechanism of adaptations under drought stress [70]. Earlier studies revealed that 20 CIPK genes are upregulated in rice, specifically under drought stress conditions. A recent RNA-seq study reported the overexpression of these CIPK genes under various abiotic stresses, such as salinity and cold [71]. RNA-seq studies reported that rice cultivars tolerant to salinity have a quick response to salinity and earlier induction of H_2_O_2_ and signal transduction than sensitive ones [72]. Salinity-tolerant cultivars set up an adaptive program by limiting sodium to roots and old leaves and activating the genes related to photosynthesis in new leaves. Two inbred lines with extreme cold tolerance and sensitivity were used for whole-genome transcriptomics, and bioinformatics analysis and the results indicated that 948 differentially expressed genes (DEGs) out of a total of 19,794 genes were mainly responsible for DNA binding, ATP binding, and protein kinase [73]. RNA-seq of drought-resistant and drought-susceptible cultivars of sorghum at seedling stage under PEG-induced drought revealed 180 DEGs; 70 genes were uncharacterized novel genes or associated with transcription factors and signal transduction under stress [74].

**Table 3:** Transcriptome profiling in five major field crops in abiotic stresses condition.

Crop	Tissue	Technique	Abiotic Stress	Reference
Rice	Leaves	RNA-Seq	Drought	[75]
Rice	Leaves	Microarray	Cold	[76]
Rice	Leaves and shoot	RNA-Seq	Adaptive and salinity	[72]
Wheat	Roots	RNA-Seq	Drought	[77]
Wheat	Crown tissue and leaves	RNA-Seq	Cold and light	[78]
Wheat	Shoots and roots	Microarray	Salinity	[79]
Maize	Tassels	RNA-Seq	Drought	[80]
Maize	Leaves	RNA-Seq	Salinity	[81]
Maize	Leaves	RNA-Seq	Cold	[73]
Barley	Leaves and roots	Microarray	Drought	[82]
Barley	Roots	RNA-Seq	Salinity	[83]
Sorghum	Seedlings	RNA-Seq	Drought	[74]
Sorghum	Seedlings	RNA-Seq	Salinity	[84]
Sorghum	Seedlings	RNA-Seq	Salinity	[85]

### 3.3. Application of Transcriptomics for Crop Improvement against Biotic Stress

Crop yield is challenged by various biotic stressors, such as bacteria, viruses, fungi, insect pests, and weeds [86]. Most plant-breeding programs target developing genotypes, which are tolerant or resistant to plant pathogens and insect pests so that crop loss due to biotic stress could be mitigated. Plants have evolved with various biochemical and physiological mechanisms to escape biotic stresses [87]. In response to pathogen infection, plants activate salicylic acid (SA), jasmonic acid (JA) and ethylene (ET) signaling, reactive oxygen species (ROS) production, hypersensitive response, the release of toxic compounds, and phytoalexins [88]. Therefore, understanding the molecular level changes in plants in response to pathogen attack is crucial to develop disease-resistant crop varieties.

Various transcriptomic studies were conducted in cereals to decipher the disease-resistance mechanisms and to identify resistance (*R*) genes. The whole-genome transcriptome analysis of four wheat cultivars, namely Wuhan 1, Nyubai, HC374, and Shaw, after head inoculation with *Fusarium graminearum*, revealed upregulation of leucine-rich repeats receptor kinases (LRR-RKs), a class of receptor kinases involved in disease resistance during different time points in resistant and susceptible cultivars. The differential expression profile of these genotypes showed various genotype-specific defense responses [89]. Table 4 summarizes some important examples where transcriptome data was used to study the plant response against various plant pathogens. *Mangnaporthe oryzae*, causing blast disease in rice, was the first pathogenic fungus to be sequenced. Hence, *Magnaporthe oryzae*-rice is considered as a model pathosystem to understand molecular host–pathogen interactions. High-quality transcriptomic studies via RNA-seq provide essential information to dig out genomic level interactions of host-pathogen systems [90]. It is well known that the *Xa23* gene in rice confers broad-spectrum resistance to most of the biotypes of *Xanthomonas oryzae* pv. *oryzae* (*Xoo*). The transcriptome profiling of NILs with *Xa23* (CBB23) and without *Xa23* (JG30) before and after inoculation with *Xoo* provides insight into the downstream genes and pathways involved in the resistance provided by the *Xa23* gene. In total, 1645 DEGs were found, and most of these are associated with phenylpropanoid biosynthesis, followed by flavonoid biosynthesis and phytohormone signaling [91].

### 3.4. Challenges and Prospects in Transcriptomics

Transcriptomic studies faced various challenges from time to time; most of these were resolved with the advancement of techniques, and some are still in the pipeline. Microarray is limited to depict the expression level of only known genes. This was overcome by RNA-seq that provides a complete profile of the transcript present at any stage or time of an organism without missing any transcript. It also lowers the background noise and increases the experiment’s clarity. Analysis of NGS data using RNA-seq is, however, time consuming because read coverage may not be uniform along the genome due to variation in nucleotide composition between genomic regions. In RNA-seq, a long transcript is estimated to have more reads than a short transcript at the same expression level. To normalize the counts with respect to transcript length, some software packages are used that represent RNA-seq data by transformed quantities, such as RPKM (Reads Per Kilobase per Million mapped reads) or the related FPKM (Fragments Per Kilobase per Million mapped reads). The software, such as Cufflinks/Cuffdiff, provides an integrated analysis pipeline from the aligned reads to the differential expression results, where the inference is based on FPKM values. Further improvements in RNA-seq are revolutionizing the transcriptomics studies in plants to develop crop varieties in the near future which can withstand biotic and abiotic stress and produce a higher yield.

## 4. Cereal Proteomics

The advances in genomic techniques provide a blueprint of possible gene products that have changed our way of studying biological systems. As the genome is static, it lacks a correlation between mRNA and protein abundance due to post-translational modifications, protein function, and localization. In addition, it does not give a biological snapshot of an organism at a particular developmental time point. Therefore, it is essential to study the protein structure, their interactions to explore their role during plant growth, and development. Proteomics is a comprehensive, high-performance approach for identifying and analyzing protein expression at a particular time and condition in a cell, tissue, or organelle of an organism [101]. The first report of 2-DE dates back to 1975, which provided the first glimpse of the protein levels and the isoforms of the cells. Marc Wilkins coined the term “proteomics” in 1994 as an extension of the word “proteome” (PROTein complement of the genOME) at the first two-dimensional electrophoresis (2-DE) meeting in Siena, Italy [102]. The study of proteome profiles provides deep insight into various metabolic processes and their interaction with different regulatory pathways in a biological system. Proteomics is a powerful tool providing robust and better representation of the cell functioning than other techniques, including genomics tools.

The advancements in proteomics in the past decades have led to new and improved technologies, such as two-dimensional polyacrylamide gel electrophoresis (2D-PAGE), liquid chromatography (LC), and mass spectrometry (MS), which have enabled fast and accurate protein identifications.

### 4.1. Technical Advances in Proteomics

**Figure 4:**
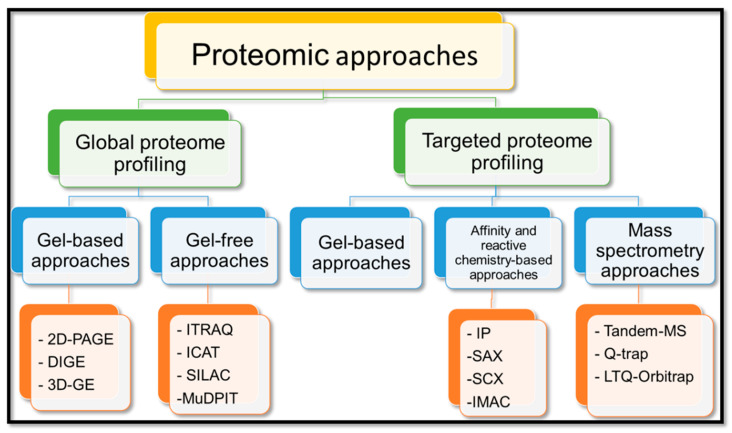
Schematic representation of various proteomics approaches.

In the recent past, various proteomics approaches have been developed and adopted in plants. These tools paved the way for high-throughput proteome analysis for quantification and localization of protein–-protein interactions, and post-translational modifications (PTMs). Most of the proteomics technologies have three main steps, including identification or quantification (mass spectrometry; MS), protein extraction, and separation (gel-based or gel-free/Column-based methods) [103]. The gel-free techniques can be label-free, such as LC coupled with MS (LC–MS), or tag-based, such as ICAT, iTRAQ, etc. [104] (Figure 4). A single technology cannot comprehensively analyze a complete plant proteome due to its complex and dynamic nature. Therefore, multiple approaches are used to improve the understanding, resolution, and coverage of plant proteome. Various factors such as resource availability, facilities, and applications, e.g., global or targeted profiling, decide the proteome’s study approaches [103].

### 4.2. Global Proteome Profiling

Global proteome profiling is considered as one of the best approaches for comparing two or more proteomes or generating a reference proteome map. Table 5 categorizes the proteome profiling into gel-based and gel-free/shotgun approaches [105]. Gel-free proteomics is gaining popularity with the passing years due to increased reproducibility and less bias than gel-based proteomics [106].

#### 4.2.1. Gel-Based Approaches

These are the most popular, versatile, and mature methods of protein separation and quantification. They allow the identification of low-abundance proteins, characterize protein isoforms on a large scale, and are less expensive than gel-free approaches.

Two-dimensional polyacrylamide gel electrophoresis (2D-PAGE) is considered the workhorse of proteomics due to its affordability and acquaintance. It is widely used in expression proteomics studies. It resolves proteins based on two independent parameters: isoelectric point (pI) and molecular mass (M). Depending upon the M, the proteins can be fractionated into two dimensions based on the presence or absence of 2-mercaptoethanol. The proteins can be resolved by staining with dyes, such as Coomassie blue, silver nitrate, or SYPRO Ruby, for their visualization.

The need to overcome the limitations of 2D-PAGE, like the gel-to-gel variation and less reproducibility, led to the development of difference in-gel electrophoresis (DIGE). In this approach, many protein samples labeled at their lysine residues by different fluorophores (CyDye2, CyDye3, CyDye5), besides Coomassie Blue, silver nitrate, or SYPRO Ruby, are simultaneously separated on a single gel [107]. DIGE is used to elucidate variations in protein expression in response to various biotic and abiotic stresses.

Three-dimensional gel electrophoresis (3DGE) advances 2D-PAGE to overcome the co-migration interferences [108]. It uses two different buffers with different ion carriers and gives very accurate protein and PTMs’ identification [109].

Mass spectrometry (MS) is used to identify proteins of interest after the extraction of peptides by in-gel digestion [110]. Various computer algorithms help in the identification of proteins on the basis of peptide mass and fragmentation (MS/MS) information. The overall process of protein identification by MS includes three steps. The transformation of molecules to gas-phase ions, separation of ions based on mass to charge ratio (*m*/*z*) in electric or magnetic field, followed by the measurement of separated ions with particular *m*/*z* value. The methods used for ionization include matrix-assisted laser desorption ionization (MALDI), surface-enhanced laser desorption/ionization (SELDI), and electrospray ionization (ESI) [111].

#### 4.2.2. Gel-Free Approaches

Gel-free approaches are developed to overcome the limitations of gel-based approaches, such as the inability to separate the entire proteome, rare detection of low-abundance proteins, and labor-intensive nature. These include quantitative approaches, like tag-based labeling (ICAT, iTRAQ), metabolic labeling (SILAC), and label-free methods (MudPIT) [105].

Isotope-Coded Affinity Tagging (ICAT) is an in-vitro isotopic labeling approach for protein quantification, which involves the use of an affinity tag (biotin), linker having stable isotope, and a reactive group that binds to thiol groups (cysteines) of proteins. The labeled tryptic peptides are first fractionated by chromatography and then identified by mass spectrometry (MS) [112]. ICAT mainly contributes to identify novel proteins controlling a vital biological function in a particular cultivar [113].

Isobaric Tagging for Relative and Absolute Quantification (iTRAQ) is a multiplex protein-quantification technique utilizing the isobaric tags for labeling the N-terminus and side-chain amine groups of proteins. The sensitivity of protein quantification from different sources in one test is much higher than ICAT [114]. Crop breeders use this technique to elucidate markers for biotic and abiotic stresses, and those later can be used in designing genetically modified crops [115].

Stable Isotope Labelling by Amino Acid in Cell Culture (SILAC) is a metabolic labeling technique that is the most potent approach for dynamic quantitative plant proteome studies. It utilizes in-vivo labeling of cell population grown in either N14 or N15 containing medium [116]. It is advantageous to identify proteome changes in signaling pathways triggered by PTMs in response to stress [117].

Multi-Dimensional Protein Identification Technology (MudPIT) is a shotgun proteomics tool used for complex multi-dimensional protein analysis [106]. It is a less complex and highly sensitive technique for the identification of low-abundance proteins. In this approach, the biphasic or triphasic microcapillary columns are used to separate digested proteins, followed by performing tandem MS. This technology has been used to unravel the mechanisms involved in controlling tiller numbers in rice [106].

### 4.3. Targeted Proteome Profiling

It is a selective proteome analysis of interacting proteins or post-transcriptionally modified proteins using PTM-specific stains, antibodies, or targeted MS assays [103]. It can be classified into gel-based, affinity and reactive chemistry-based, and MS-based targeted proteomics.

#### 4.3.1. Gel-Based Proteomics

The global proteome analysis is undertaken using 2D-PAGE, followed by staining with Phosphoprotein specific gel stain (Pro-Q Diamond; PTM specific stain). However, these approaches are not used these days due to a lack of identification of less abundant proteins [103].

#### 4.3.2. Affinity and Reactive Chemistry-Based Proteomics

In this approach, specific proteins are isolated, enriched, and purified by different techniques, such as immunoprecipitation (IP), strong cation exchange (SCX), strong anion exchange (SAX), and immobilized metal affinity chromatography (IMAC). These techniques can be used individually or coupled with one another to enhance efficiency.

#### 4.3.3. MS-Based Proteomics

They are based on detecting signals resulting from transitions in the ions during the fragmentation in the mass spectrometer. Various tools, like tandem MS, Quadrupole Trap (Q-trap), triple quadrupole, and Linear Trap Quadrupole Orbitrap (LTQ-Orbitrap), are commonly used. Selected Reaction Monitoring (SRM) is the process of detection of transitions in triple quadrupole, whereas Multiple Reaction Monitoring (MRM) is the detection of multiple modifications [118]. However, the aforementioned techniques suffer from precision errors between samples. To overcome this shortcoming, SRM/MRM techniques are isotopically labeled [119]. Table 6 provides knowledge about different proteomics techniques used to study abiotic and biotic stress responses in cereal crops, including wheat, barley, rice, maize, and sorghum.

Although the field of MS has advanced enormously, still, there are many shortcomings, including lower detection limits and limited coverage of proteome in the characterization of complex biological samples [139]. This technique demands very careful handling of samples, as certain protease inhibitors can change pI and electrophoretic mobility of the proteins [140]. Many technical replicates are required for the analysis due to the poor reproducibility and low accuracy. MS-based techniques are not sensitive enough in order to identify the low-abundant proteins in a sample. Future developments in MS and identification methods will overcome these limitations.

### 4.4. Peptidomics, Phosphoproteomics, and Redox Poteomics

The vast variety of novel peptides, e.g., the ones derived from non-functional precursors, functional precursors, and not derived from a precursor protein cannot be characterized by standard analytical methods using MS. Peptidomics is the identification and comprehensive analysis of these physiological and pathological peptides smaller than 10-kDa in size [141]. In this technique, the native peptides are used as such and are not subjected to chemical or enzymatic cleavage. It has been utilized in plants like *Arabidopsis thaliana* and Medicago.

Phosphorylations characterized by protein kinases (PKs) change the protein functions such as enzyme activity, protein–protein interactions, subcellular localization, etc. Phosphoproteomics is a technique for identification of uncharacterized PKs and their substrates. There are three databases, namely PhosPhat, Pep2Pro, and PepBase, that include information of plant phosphoproteome [142].

Redox proteomics is defined as the identification of PTMs involved in all stages of plant development as a result of protein oxidoreduction, which helps in finding protein damage due to the oxidative stresses [143]. The dynamic nature of redox PTMs is the major challenge in the development of this technique. The continuous development of protein MS instruments along with quantitative proteomics will lead to the new possibilities in these areas of proteomics.

### 4.5. Bioinformatics in Proteomics

The technical advances in proteomics approaches have made it possible to achieve a massive amount of high-quality protein-expression data. It is challenging to associate this data with other omics technologies, like genomics, transcriptomics, metabolomics, and phenomics. Bioinformatics tools play a fundamental role in overcoming this bottleneck by reducing the analysis time and providing statistically significant results. Some of the major proteomics databases currently used are PRoteomics IDEntification database (PRIDE) [144], Peptide Atlas [145], and Mass Spectrometry Interactive Virtual Environment (MassIVE). Various comprehensive databases for plant proteomics, such as Plant Proteomics Database (PPDB), 1001 Proteomes, Pep2 Pro Database, DIPOS, etc. [146,147,148,149], as well as different web-based prediction tools, like GelMap [150], MRMaid [151], Peptide Atlas SRM Experiment Library (PASSEL) [152], etc., have been developed to assist proteome analysis.

### 4.6. Challenges and Prospects in Proteomics

The proteomic analysis complements both transcriptomics and metabolomics for elucidating plants’ cellular mechanism and thus is a vital tool for crop improvement. The recent advancements in proteomics techniques have enabled us to unravel plant biology. However, we still need to overcome the various limitations of these techniques to develop smart crops with high grain quality capable of withstanding multiple stresses. New emerging technologies, such as peptidomics, phosphoproteomics, and redox proteomics, will provide in-depth insight into molecular interactions and protein function [153]. With the ever-changing climate, new plant variants are being introduced continuously to cope with ambient fluctuations. Novel proteomic tools will enable us to generate more stress-tolerant or stress-adaptive cultivars.

## 5. Cereal Metabolomics

Metabolomics is a relatively new “omics” technology for deciphering the plant metabolomes and understanding complex biological systems. Metabolomics allows comprehensive profiling and comparison of small molecules (<1500-Da) of a cell, tissue, organ, or organism [154]. Metabolomics deals with identifying and quantifying metabolites in a biological system to investigate their compositions and interactions with the environment [155].

Based on the purpose of the study, metabolomics can be differentiated into two types, namely targeted and untargeted. Targeted metabolomics deals with the absolute quantification of one or a few metabolites in a set of predefined known substances. Therefore, the targeted approach tends to be highly sensitive and quantitative and can be helpful to trail the metabolites known to be associated with specific stress. Thus, targeted metabolomics is a discovery-based approach and measures the relative abundances of several hundred to thousands of all detectable metabolites. The untargeted approach, on the other hand, can measure mass spectrometric features of unknown metabolites and thus enhance the chances of sensing unintended effects [155].

In recent years, metabolomics has been used to understand biotic and abiotic stresses in crop plants, and many studies summarize the metabolomic advances in maize, sorghum, wheat, rice, and barley, investigating the composition of these crops and/or their products and their applications for crop improvement (reviewed in [156,157,158]). Understanding the plant metabolomic processes would be beneficial for improving crop yield and human nutrition aspects in crop-breeding programs.

### 5.1. Overview of Metabolomic Pipeline

The workflow for metabolomics involves a series of steps, including experimental design, sample preparation and extraction, metabolite detection using analytical techniques, and data processing and analysis using bioinformatics techniques. Since metabolomics involves a wide range of diverse compounds, variations in metabolite concentration (~106) can complicate the downstream analyses [159]. Thus, it is essential to carefully choose (1) the appropriate experimental design, (2) optimized sample preparation and extraction protocols, and (3) detection technologies for comprehensive metabolomic analyses.

Numerous extraction protocols for metabolomics analysis [160,161] are available, and optimizing the metabolomic protocol is an essential step in metabolomics [159]. For example, targeted metabolomics can be optimized to increase the signal-to-noise (s/n) ratio of the desired metabolite or decrease the time and cost of experimentation [155]. The untargeted approach, on the other hand, must be optimized for reproducibility of the protocol to detect the ratio of the actual variation in a biological sample to the variation due to experimental errors. Several approaches, such as fractional factorial analysis or D-Optimal design to experimental design, can optimize metabolomic protocols [155].

### 5.2. Analytical and Data Processing Techniques in Crop Metabolomics

Several techniques, such as gas chromatography mass-spectrometry (GC–MS) [162], liquid chromatography-mass spectrometry (LC–MS) [163], capillary electrophoresis mass spectrometry (CE–MS) [164], nuclear magnetic resonance (NMR) [165], and vibrational spectroscopy (VS) [155], have been applied in crop metabolomic studies. With recent advancements in technology, other methods, such as gas chromatography time-of-flight mass spectrometry (GC–TOF–MS) [166], ultra-performance liquid chromatography-mass spectrometry (UPLC–MS) [167], capillary electrophoresis time-of-flight mass spectrometry (CE–TOF–MS) [168], high-performance liquid chromatography (HPLC) [169], and liquid chromatography high-resolution mass spectrometry (LC–HRMS) [170], have been utilized in crop metabolomic studies. Table 7 provides an overview of commonly used analytical techniques in crop metabolomics.

After analytical analyses with one or more of the techniques mentioned above, the data then undergoes a series of pre-processing steps, including cleaning, noise reduction, baseline correction, alignment, peak deconvolution, normalization, and scaling. Numerous online platforms have been developed to help metabolomics, data mining, data assessment, data processing, and data interpretation. Statistical analyses, e.g., principal component analysis (PCA), multivariate curve resolution (MCR), hierarchical cluster analysis (HCA), partial least squares discriminant analysis (PLS-DA), and batch-learning self-organizing map (BL-SOM), are commonly used to make meaningful inferences from large metabolomics datasets [156,171,172]. After profiling metabolites in a particular plant species, metabolic pathways can be reconstructed from a list of functionally annotated genes available from the databases, such as KEGG pathway or KNApSAcK [173,174].

### 5.3. Applications of Metabolomics for Crop Improvement

Metabolomics has widely been used to investigate the plant’s adaptive responses against stresses. It plays an essential role in investigating the synthesis of specific metabolites under various stresses to understand how plants adapt to unfavorable surroundings. Metabolomic studies uncover new compounds and novel metabolic pathways that accumulate under different stress conditions [158]. Besides, metabolomics studies also help in improving the understanding of previously recognized metabolic pathways. Over the last decade, several metabolome studies have been conducted to investigate the metabolite concentration changes under various biotic and abiotic stress factors, as described in Table 8.

The drought-stress response has also been studied by metabolomic approaches in rice [162], wheat [167], maize [166], and sorghum [175]. Variations in phytohormones and other metabolites in the roots of barley plants under salinity stress were reported [145]. In rice, profiles of flavone-glycosides, which are major secondary metabolites, were evaluated against abiotic stress and herbivores [176]. Researchers have reported natural metabolic variations in rice [177]. Moreover, identifying the metabolites encoding for specific loci can potentially be utilized as biomarkers in association studies [158]. Metabolome Quantitative Trait Loci (mQTLs) analysis investigates metabolite concentrations in plant tissues (m-trait) and can, therefore, provide a comprehensive understanding of their genetic background. Furthermore, mQTL can discover novel relationships between metabolomic pathways, structural genes, and agronomically important traits; hence, it can assist in crop breeding; An example being a comparative mQTL mapping between rice and maize [160].

**Table 8:** Examples of studies investigating the crop response to biotic and abiotic stresses using metabolomics techniques.

Crop	Stress	Techniques	References
**Abiotic stresses**			
Rice	Flooding	GC–MS, NMR	[178]
Rice	Drought	GC–MS	[162]
Rice	Low temperature	LC–MS/MS	[179]
Wheat	Drought	UPLC–MS	[167]
Wheat	Low nitrogen	UPLC–QTOF–MS	[180]
Maize	Salinity	NMR	[181]
Maize	Drought	GC–TOF–MS	[166]
Maize	Low nitrogen	GC–MS	[182]
Barley	Salinity	LC–MS	[163]
Barley	Drought	GC–MS	[183]
Sorghum	Drought	GC–MS	[175]
Sorghum	Low nitrogen	GC–MS/LC–MS	[184]
**Biotic stresses**			
Rice	*Magnoporthe grisea*	NMR, GC/LC–MS/MS	[165]
Rice	*Rhizoctonia solani*	GC–MS	[168]
Wheat	*Stagonospora nodorum*	GC–MS	[185]
Maize	*Fusarium verticillioides*	LC–HRM	[186]
Barley	*Fusarium graminearum*	HPLC, LC–HRMS	[169]
Barley	*Fusarium graminearum*	LC–MS	[173]
Sorghum	*Burkholderia andropogonis*	LC–MS	[174]

### 5.4. Challenges and Prospects in Crop Metabolomics

Integrating metabolomics with the genetic approaches can facilitate studying the genetic regulation of plants in relation to metabolomics. Furthermore, utilizing high-throughput genome sequencing, reverse genetics with metabolomics tools can decrease the time, such as in metabolomics-assisted breeding. These novel plant-breeding approaches can thus help crop improvement programs produce high-yielding crops, stress-tolerant germplasm, and climate-adapted crop varieties.

Prospects of metabolomics include screening the metabolic markers to understand plant metabolism. Emerging technologies, such as single-cell metabolomics with metabolome-scale labeling, will improve metabolite interpretation, metabolic pathway elucidation, and metabolite quantification at the single-cell level [187]. Recent technological advancements, such as the single-probe MS technique, have the potential for near in situ targeted metabolomic analyses with minimum cell manipulation at the cellular level [188]. Future challenges of metabolomics would be to better utilize the available information from metabolomics and interpret the metabolite information correctly for possible applications.

## 6. Cereal Phenomics

During the last two decades, genomics has revolutionized plant breeding mainly due to a reduction in genotyping costs, which results in the adoption of new technologies, such as linkage mapping, genome-wide association studies, genome-wide selection, and rapid generation advance [189]. Accurate genetic mapping and genome-wide selection require precise phenotyping of the plants. However, plant phenomics, i.e., applying tools and methodologies to study plant growth, development, performance, and composition, is a field that is still in its infancy and, therefore, has lagged in comparison to genomics [190]. Since the conventional field phenotyping employed by most plant breeders is labor intensive, costly, and subjective [191], plant phenomics is a rapidly expanding domain that ranges from high-throughput field phenotyping to cellular-level imaging. Nevertheless, during the last decade, more focus was given to field-based high-throughput phenotyping (HTP), primarily to predict agronomic and physiological traits [192]. In this regard, HTP has demonstrated its potential for non-destructive phenotyping of the various agronomic, physiological, as well as biotic and abiotic stress-related traits [193] via (1) utilizing high-throughput tools and platforms, (2) image processing and implementing algorithms for the extraction of raw data, and (3) linking to the processed data to the target traits [194].

Various aerial or ground-based HTP platforms have been developed for measuring different plant traits at different growth stages with more precision, throughput, and accuracy [195]. Table 9 provides various phenotyping platforms and their use in rice, wheat, maize, barley, and sorghum. The development of novel imaging sensors for non-invasively phenotyping a wide range of organs, tissues, and physiological processes has provided a substantial impetus to the HTP [196]. This section of the review concentrates on (1) various phenotyping platforms that are currently being used to accelerate genetic gains in key cereals, (2) advancements in imaging sensors and subsequent analyses, and (3) application of machine and deep-learning methods for solving the “big data” problems in phenomics.

**Table 9:** List of phenotyping platforms and their utilization.

Phenotyping Platform/ Techniques	Utilization	References
BreedVision	Tractor-pulled multisensory phenotyping platform with RGB, multispectral, and time-of-flight sensors	[197]
GROWSCREEN fluoro	Work under controlled conditions for quantification of fluorescence pigments	[198]
Light curtain analysis	Utilized for leaf area and plant height estimation	[199]
LEAF-E	Estimates the total leaf growth and rate of development	[193]
Phenocart	A movable platform in the field used for high-throughput phenotyping	[192]
Phenopsis	Used to study drought tolerance abilities under control conditions	[200]
Phenoplant	Used to obtain chlorophyll fluorescence parameters under controlled conditions	[201]
Phenovator	Used for phenotyping a large number of samples under controlled conditions by providing fluorescence, multispectral, and RGB images	[202]
Pushcarts	Carts with different sensors used to study plant response to drought, heat, and other stresses; operated by one person	[190]
Terrestrial laser scanning	Used for measuring plant height and architecture under field conditions	[203]
TRiP	Used to study circadian changes in plants with a series of images and TrRiP algorithm	[204]
Unmanned aerial platforms	Multiple sensors can be employed for measuring various traits throughout the field	[205]

### 6.1. Plant Phenotyping Platforms

HTP depends on the imaging sensor used. Advanced phenotyping platforms have improved the data-capture capabilities by including mobility, throughput, and inbuilt data storage at a relatively low cost. Unmanned aerial vehicles (UAVs) have maximum adoption due to their reliability, cost, and technical requirements; however, some countries are still not adopting it due to regulations controlling their flights. Several carts and tractor-mounted tools have similarly been adopted for various crops, although their utilization is also stage dependent [190]. Moreover, several handheld cheap platforms provide spectral and time-series information. However, these handheld devices face standardization and low-throughput issues; because they are usually mounted over poles, they result in less canopy coverage [197]. Table 9 provides detailed information about various platforms utilized during the last decade.

### 6.2. Imaging Sensors and Analysis

Imaging sensors have enabled the collection of high-resolution and multidimensional data from plants to quantify plant growth, yield, stress, and physiological process under both control and field conditions. The recent development of sensor technology measuring reflection from gamma rays to radio waves regions of the electromagnetic spectrum has provided a plethora of information to plant scientists. These imaging sensors vary from spectroscopy, sound navigation ranging (SONAR), light detection and ranging (LIDAR), X-ray computed tomography (CT), thermal, visible to near-infrared, multispectral, hyperspectral, fluorescence, time of flight (ToF), positron-emission tomography, and stereovision [202,204]. The utilization of these imaging sensors with autonomous platforms has opened up the doors of HTP. Table 10 and Table 11 provide detailed information about different imaging sensors utilized for studying agronomic traits and biotic and abiotic stresses in the five most important crops grown in the world: rice, wheat, maize, barley, and sorghum.

#### 6.2.1. RGB/Visible Imaging

RGB cameras or regular cameras or digital cameras capture the true color images in the electromagnetic spectrum’s visible region. This is the cheapest and most often used sensor for plant phenotyping studies. These sensors reflect the red, green, and blue regions of the visible spectrum. It has been used to estimate plant biomass, different pigments, tiller count, yield traits, flowering time, biotic stresses, plant height, germination, and emergence rates [206,207].

#### 6.2.2. Multispectral Imaging

Multispectral cameras provide information about specific wavelength bands from the spectrum’s visible and infrared regions. These reflection bands are used to extract different vegetation bands, which give information about photosynthetic efficiency, pigments, nutrient status, water status, and plant senescence [208]. The essential indices utilized include normalized differentiation vegetation index (NDVI), water index (WI), anthocyanin reflection index (ARI), and simple ratio (SR) [209].

#### 6.2.3. Hyperspectral Imaging

These imaging sensors cover whole visible and infrared regions with a high spatial resolution by covering reflection from the entire areas due to the sensor’s small bandwidth. These sensors have the best spatial and spectral resolution, resulting in more useful information. This imaging platform has been used for studying plant health status, leaf growth, predicting grain yield, biotic stresses, water status, plant height, and chlorophyll content [209,210].

#### 6.2.4. Thermal Imaging

These sensors provide information about plant water status by measuring reflection from the infrared region for estimating canopy temperature and transpiration rate. Thermal imaging has been used for detecting plant water status, disease-infected plants, and the maturity of the kernels [211,212].

#### 6.2.5. Fluorescence Imaging

Fluorescence sensors provide information about photochemistry changes by capturing photosystem II’s fluorescence emissions. Plants absorb a specific portion of the electromagnetic spectrum and thus have a characteristic emission spectrum. Fluorescence sensors provide information about the photosynthesis rate, chlorophyll content, and various physiological processes in plants [213].

#### 6.2.6. X-ray Computed Tomography

These imaging sensors aid in generating 3D tomographic images of the objects using an extensive series of 2D radiographic images taken with computer-processed X-rays. Images provide root architectures by separating objects depending on the different densities. X-ray CT has been utilized for studying root traits, tillers morphology, and grain quality [194,214].

In addition to all these imaging sensors, there are several others: positron-emission tomography, magnetic resonance imaging, SONAR, laser scanning, LIDAR, and flight time. For details of sensor readings, the readers are referred to other publications [143,194,209,215,216].

**Table 10:** Application of high-throughput phenotyping platforms and imaging sensors for improving abiotic stresses and agronomic traits in field crops during the last decade.

Crop	Phenotyping Platform Sensor or Techniques	Field/ Lab	Abiotic Stresses/ Agronomic Traits	Imaging Sensor	Description	Reference
Rice	Ground-based platforms	Lab	Salinity	Thermal imaging	Plant growth and transpiration rate was used to predict the salinity responses of plants	[214]
Rice	Ground-based platforms	Field	Nitrogen content	Hyperspectral imaging	Reflectance information and cumulative temperature data were used in the partial least square method for predicting nitrogen status	[210]
Rice	Ground-based platforms	Field	Drought stress	RGB imaging	Stay green-related feature were extracted for assessing drought-tolerance ability	[196]
Wheat	Ground-based platforms	Field	Drought	Passive and active hyperspectral reflectance sensors	Performances of different sensors were evaluated for predicting drought tolerance abilities of genotypes with water stress indices	[208]
Wheat	Manned helicopter	Field	Water and heat stress	Thermal imaging	Canopy temperature was measured in high-throughput way for avoiding the plot-to-plot variation with handheld infrared thermometers	[212]
Wheat	Ground-based platforms	Field	Nitrogen content	Hyperspectral imaging	Leaf nitrogen status was measured from spectral information with a calibrated model	[217]
Maize	Organ/tissue phenotyping	Lab	Drought stress	Hyperspectral imaging	Support vector machine classification method separated the water-stressed genotypes from healthy plants with information from vegetation indices	[218]
Maize	Unmanned aerial vehicle	Field	Water status in plants	Multispectral and thermal imaging	Crop water stress index was predicted from the multispectral images to decipher the plant water status	[219]
Maize	Unmanned aerial vehicle	Field	Weeds	RGB imaging	Loss of greenness from maize was used for separating weeds from the plants	[220]
Barley	Ground-based platforms	Field	Drought	Hyperspectral imaging	Linear ordinal support vector machine model was used to predict the drought responses in the plants	[209]
Barley	Organ/tissue phenotyping	Lab	Salinity	Thermal imaging	Infrared imaging was used to differentiate salt concentration among the genotypes	[191]
Barley	Unmanned aerial vehicle	Field	Nitrogen use efficiency	RGB, multispectral, and thermal imaging	UAV’s having RGB, multispectral, and thermal imaging was utilized for nitrogen use efficiency	[221]
Sorghum	Ground-based platforms	Field	Plant height	RGB, ultrasonic, and LIDAR sensor	A comparison was performed for predicting sorghum height, with the LIDAR sensor performing best	[222]
Sorghum	Unmanned aerial vehicle	Field	Drought stress	RGB imaging	Plant height, biomass, and leaf area were measured for assessing the drought-tolerant abilities of genotypes	[223]

**Table 11:** Application of high-throughput phenotyping platforms and imaging sensors for improving biotic stresses in field crops during the last decade.

Crop	Phenotyping Platform/Sensor/Techniques	Field/Lab	Disease/Pest/ Virus	Imaging Sensor	Description	References
Rice	Ground and aerial platforms	Field/ Lab	Rice blast	Multispectral imaging	Reflectance values were correlated with the disease severity	[224]
Rice	Organ/tissue phenotyping	Lab	Alfatoxin	Near-infrared spectroscopy	Partial least regression utilized reflectance information for separating infected and healthy seeds	[225]
Rice	Unmanned aerial vehicle	Field	Rice sheath blight	RGB and multispectral imaging	Percentage of infected leaves from RGB images and vegetation indices from multispectral imaging aid in the detection of rice sheath blight	[226]
Wheat	Ground-based platforms	Field	*Septoria tritici* blotch	Hyperspectral imaging	Spectral reflectance indices derived from hyperspectral imaging aids in detecting the presence and severity of Septoria tritici blotch	[189]
Wheat	Organ/tissue phenotyping	Lab	*Fusarium* head blight	Hyperspectral imaging	Fusarium head blight was detected using visible-NIR imaging of wheat grain, and grains were separated using linear discrimination and principal component analysis	[227]
Wheat	Unmanned aerial vehicle	Field	Yellow rust	Hyperspectral imaging	Deep convolutional neural network utilizing both spectral and spatial resolution provided the best performance for predicting yellow rust	[228]
Maize	Ground and aerial platforms	Field	Northern leaf blight	RGB imaging	A convolutional neural network was used for classifying the infected leaves	[229]
Maize	Organ/tissue phenotyping	Lab	Alfatoxin infection	Fluorescence imaging	Discriminant analysis from the imaging data aids in the separation of healthy and affected kernels	[213]
Maize	Unmanned aerial vehicle	Lab	Tar spot	Multispectral and thermal imaging	Disease-progression curve was analyzed using vegetation indices derived from the images	[230]
Barley	Ground-based platforms	Field	Powdery mildew	Hyperspectral imaging	Support vector machine was used for early detection of disease symptoms by measuring reflection bands	[231]
Barley	Ground-based platforms	Field	Blast	Hyperspectral imaging	Spectral angle mapping and spectral unmixing analysis was used to locate the pathogen lesions	[232]
Barley	Organ/tissue phenotyping	Lab	Rust and powdery mildew	Hyperspectral imaging	A simple volume maximization algorithm was developed for differentiating different infected leaves	[233]

### 6.3. Challenges and Prospects in Crop Phenomics

The continuous use of aerial and ground-based HTP platforms with different imaging sensors at multiple points during different growth stages of the plants has resulted in big data, storage issues, and the extraction of valuable information. This issue is resolved by adopting machine and deep-learning tools for data analysis to extract legitimate conclusions from the big data sets [194,234]. Machine learning (ML) is an interdisciplinary approach for data analysis using probability, statistics, classification, regression, decision theory, data visualization, and neural networks to relate information extracted with the phenotype obtained. ML provides a significant advantage to the plant breeders, pathologists, and agronomists to extract many parameters for analyzing each trait together, despite traditional methods in which we used to look at a single feature at a time [235]. The other great breakthrough with ML is directly linking the variables extracted from the HTP data to the plant stresses, biomass accumulation, grain yield, and soil characteristics [223,236]. ML’s most significant success involves inferring trends from the data and generalizing the results by training the model. There have been various ML models being applied for HTP, namely support vector machine [220], discriminant analysis [227], k means clustering [209], neural network [211], clustering [209], and dimensional reduction [194]. All these models help identify, classify, quantify, and predict different phenotyping components in plants.

However, the recent transformation by deep learning (DL) in other fields, such as traffic signaling, health care, voice and image recognition, consumer analytics, and medical diagnostics, has provided a new tool to plant scientists for image analysis in HTP [236]. DL models involve automatically learning the pattern from the extensive data set using non-linear activation functions for making conclusions, such as classification or predictions. The important DL models used for phenomics include but are not limited to a multilayer perceptron, generative adversarial networks, convolutional neural network, and recurrent neural network [229]. These potential data analysis tools aid in broadening the prospectus of HTP in plant breeding.

## 7. Conclusions

Combining the omics technologies, such as genomics, transcriptomics, proteomics, metabolomics, and phenomics, can help investigate the genetic and molecular determinants and complex pathways in cereal crops. Protein and metabolic profiling coupled with genome-wide scans can be utilized to efficiently select desirable agronomic traits, thus opening new opportunities to enhance crop yields and resistances. Furthermore, high-throughput, phenomics-enabled genome-wide mapping combined with metabolic and gene-expression studies can help explore the environmental effects of crops’ phenotypic plasticity under various biotic and abiotic conditions.

## Figures and Tables

**Table 1:** Examples of some traits studied via genome-wide linkage mapping (GWLM) and genome-wide association studies (GWAS) in five major cereals.

Crop	Mapping Method	Trait or Gene Studied	Reference
Rice	GWLM and GWAS	Seed vigor	[29]
Rice	GWLM and GWAS	Bacterial blight-resistant gene, *Xa43*(t)	[30]
Rice	GWLM and GWAS	Grain shape and grain weight	[31]
Rice	GWAS	Plant architecture	[32]
Rice	GWAS	Salt tolerance, *OsSTL1* and *OsSTL2*	[33]
Wheat	GWLM	Plant height and yield	[34]
Wheat	GWLM	Grain shape and size	[35]
Wheat	GWLM	Reduced plant height gene, *Rht24*	[36]
Wheat	GWAS	Floret fertility, assimilate partitioning, and spike morphology traits	[37]
Wheat	GWAS	Total spikelet number	[38]
Maize	GWLM	Resistance to northern leaf blight	[39]
Maize	GWLM and GWAS	Plant and ear height	[40]
Maize	GWLM and GWAS	Male inflorescence size	[41]
Maize	GWAS	Lipid biosynthesis	[42]
Maize	GWAS	Root morphology traits	[43]
Barley	GWLM	Plant height	[44]
Barley	GWLM	Awn length	[45]
Barley	GWAS	Photoperiod response	[46]
Barley	GWAS	Nitrogen use efficiency	[47]
Barley	GWAS	Spikelet number and grain yield	[48]
Sorghum	GWLM	Plant height, node number, panicle length, flag leaf length, and flag leaf width	[49]
Sorghum	GWLM and GWAS	Grain quality traits	[50]
Sorghum	GWAS	Plant architecture traits (e.g., tiller number, panicle length, seed number, internode length)	[51]
Sorghum	GWAS	Kernel composition	[52]
Sorghum	GWAS	Grain size	[53]

**Table 2:** Comparison of next-generation sequencing platforms used for RNA-Sequencing.

Platform	Read Length (in bps)	Chemical Reaction	Amplification Method	Read Pair	Overall Error Rate
**1st generation**					
Sanger sequencing	750	Chain termination	PCR	Yes	—
**2nd generation**					
454 Roche	400	Pyrosequencing	Emulsion PCR	Yes	0.5%
HiSeq Ilumina	150–300 (paired end)	Reversible termination	Solid-phase PCR	Yes	0.2%
SOLiD	75 (single-end) or 50 (paired-end)	Sequencing by ligation	Emulsion PCR	Yes	0.1%
Ion torrent	200–400	Proton detection	Emulsion PCR	Yes	1%
**3rd generation**					
PacBio	25 kb (single-end)	Real-time sequencing	Real-time single- molecular template Hi-Fi	No	0.1%
Oxford Nanopore	30 kb	Disruption of ionic current flow through nanopores	Not required	No	3%

**Table 4:** Transcriptome profiling of five major field crops under biotic stresses.

Crop	Tissue	Biotic Stress	Reference
Rice	Leaves	*Magnaporthe oryzae*	[90]
Rice	Leaves	*Xanthomonas oryzae* pv. *oryzae*	[91]
Rice	Leaf sheath	*Rhizoctonia solani*	[92]
Wheat	Spikes	*Fusarium graminearum*	[89]
Wheat	Seedlings	*Puccinia triticina*	[93]
Wheat	Leaves	*Puccinia striiformis* f. sp. *tritici*	[94]
Maize	Leaves	*Cercospora zeae-maydis*; *Cercospora zeina*	[95]
Maize	Leaves	*Fusarium graminearum*	[96]
Maize	Leaves	Maize Iranian mosaic virus	[97]
Barley	Leaves	*Ramularia coolo-cygni*	[98]
Barley	Leaves	*Blumeria graminis* f. sp. *hordei*	[99]
Barley	Leaves	*Rhynchosporium secalis*; *Cochliobolus sativus*	[100]

**Table 5:** Description of commonly used proteomics techniques.

Technique	Application	Advantages
2D-PAGE	● Protein separation ● Expression profiling	● Information about post-translational modifications (PTM) ● Relatively quantitative
DIGE	● Separation of proteins ● Quantitative expression profiling	● Higher sensitivity as compared to 2D-PAGE ● Less gel-to-gel variability ● Multiplexing
3D-GE	● Protein separation ● Quantitative expression profiling	● Overcome co-migration interferences ● High reproducibility
ICAT	● Chemical isotope labelling for quantitative proteomics	● High sensitivity and reproducibility ● Detects low abundant proteins
iTRAQ	● Isobaric tagging of proteins	● High reproducibility ● Multiplexing ● High throughput
SILAC	● Isotopic labelling of cells ● Differential expression studies	● Simple and straightforward quantitation ● Highly sensitive ● Robust ● Degree of labelling is high
MuDPIT	● Identification of protein –protein interactions	● Large protein complex identification

2D-PAGE = Two-dimensional polyacrylamide gel electrophoresis, DIGE = Difference in-gel electrophoresis, 3D-GE = Three-dimensional gel electrophoresis, ICAT = Isotope-coded affinity tagging, iTRAQ = Isobaric Tagging for Relative and Absolute Quantification, SILAC = Stable Isotope Labelling by Amino Acid in Cell Culture and MuDPIT = Multi-Dimensional Protein Identification Technology.

**Table 6:** Application of various proteomics technologies in studies of field crops response to abiotic and biotic stresses in the last decade.

Crop	Abiotic/Biotic Stresses	Techniques	References
Rice	Drought	LC–MS/MS	[120]
Rice	Bakanae disease	TMT–MS	[121]
Rice	Bacterial blight	2DE/MudPIT, MALDI–TOF/MS	[122]
Wheat	Drought	2D-PAGE	[123]
Wheat	Drought	2DE, MALDI–TOF–TOF–MS	[124]
Wheat	Yellow rust	nanoLC ESI–MS/MS	[125]
Wheat	Tan spot	2D-PAGE	[126]
Maize	Salinization	iTRAQ, LC–MS/MS	[127]
Maize	Heavy metal	iTRAQ, LC–MS/MS	[128]
Maize	Ear rot disease	iTRAQ	[129]
Maize	Maize rough dwarf disease	LC–MS/MS, TMT labeling	[130]
Barley	Drought	DIGE and LTQ-Orbitrap	[131]
Barley	Salinization	2D-PAGE	[132]
Barley	Leaf rust	LC–MS/MS	[133]
Barley	*Fusarium* head blight	2D-PAGE, MS	[134]
Barley	Powdery mildew	LC–MS	[135]
Sorghum	Heavy metal toxicity	2D-PAGE	[136]
Sorghum	Drought	DIGE	[137]
Sorghum	Downy mildew	2D-PAGE, MLADI–TOF/MS	[138]

**Table 7:** Description of commonly used analytical techniques in metabolomics studies.

Technique	Description	Advantages
LC–MS	Allows profiling of secondary metabolites, such as alkaloids, flavonoids, and phenylpropanoids, based on their different partitioning coefficients between the mobile phase (solvent) and stationary phase (column)	● Enables detection of metabolites without prior derivatization ● Useful for both reactive and thermally stable metabolites ● High sensitivity to ionized metabolites ● High mass accuracy allows the identification of unknown compounds ● A larger sample, such as 1–50 mL, can be used
CE–MS	Detect and separate polar or charged metabolites, such as inorganic ions, organic acids, amino acids, vitamins, nucleotides and nucleosides, thiols, carbohydrates, and peptides, based on their charge and size	● Allow rapid analyses with higher resolution than in LC ● Allow separation of polar or charged metabolites, which are incompatible with LC and GC ● Can use heterogeneous samples ● Easy sample preparation than in GC and LC ● Low reagent use and low cost ● Less quantity of sample, up to 1 uL can be used
GC–MS	Allow the simultaneous separation and detection of many volatile, thermally stable compounds and primary metabolites, such as sugars, amino acids, organic acids, and polyamines in complex mixtures	● High resolution ● High sensitivity to non-polar and volatile metabolites ● Lower cost than LC–MS
NMR	Record the absorption and re-emission energy of atom nuclei due to differences in an external magnetic field	● Allow detection of unknown metabolites ● Less biased and lower experimental error than in MS-based methods ● Easy sample preparation than in MS methods ● Excellent compound coverage ● Less destructive sampling ● Highly utilized in untargeted metabolomics profiling
VS	Measures slight differences in vibrational behavior of organic functional groups and chemical bonding under electromagnetic (EM) radiation	● Non-destructive method ● Minimal to no sample preparation ● Excellent compound coverage ● Untargeted metabolomic profiling with high accuracy ● High reproducibility

## Data Availability

Data are contained within the article.
